# Modulation of hippocampal dopamine metabolism and hippocampal-dependent cognitive function by catechol-O-methyltransferase inhibition

**DOI:** 10.1177/0269881112454228

**Published:** 2012-12

**Authors:** LM Laatikainen, T Sharp, DM Bannerman, PJ Harrison, EM Tunbridge

**Affiliations:** 1 Department of Psychiatry, University of Oxford, Oxford, UK; 2 Department of Pharmacology, University of Oxford, Oxford, UK; 3 Department of Experimental Psychology, University of Oxford, Oxford, UK

**Keywords:** COMT, dopamine, noradrenaline, hippocampus, memory, novelty preference

## Abstract

Catechol-O-methyltransferase (COMT) catabolises the catecholamine neurotransmitters and influences cognitive function. COMT modulates dopamine levels in the prefrontal cortex and its action in this region is generally invoked to explain its effects on cognition. However, its role in other brain regions important for cognitive function remains largely unexplored. Here, we investigated COMT’s impact on dopamine metabolism in the hippocampus and hippocampal-dependent behaviour. We examined the acute effects of a centrally-acting COMT inhibitor, tolcapone (30 mg/kg i.p.), on dopamine metabolism in the rat dorsal hippocampus, assessed both in tissue homogenates and extracellularly, using *in vivo* microdialysis. Additionally, we investigated the effect of tolcapone on delayed-rewarded alternation and spatial novelty preference, behavioural tasks which are dependent on the dorsal hippocampus. Tolcapone significantly modulated dopamine metabolism in the dorsal hippocampus, as indexed by the depletion of extracellular homovanillic acid (HVA) and the accumulation of dihydroxyphenylacetic acid (DOPAC). Tolcapone also improved performance on the delayed-rewarded alternation and spatial novelty preference tasks, compared to vehicle-treated rats. Our findings suggest that COMT regulates dorsal hippocampal neurochemistry and modulates hippocampus-dependent behaviours. These findings support the therapeutic candidacy of COMT inhibition as a cognitive enhancer, and suggest that, in addition to the prefrontal cortex, the hippocampus might be a key region for mediating these effects.

## Introduction

The catechol-O-methyltransferase (COMT) enzyme metabolises the catecholamine neurotransmitters and regulates dopamine levels in the prefrontal cortex (Tunbridge et al., 2004; Yavich et al., 2007; Kaenmaki et al., 2010). The human COMT gene contains a functional polymorphism in its sequence (Val^158^Met; rs4680), which alters enzyme activity; the rodent form of COMT lacks this polymorphism and has enzyme activity similar to the human high activity Val^158^ allele (Chen et al., 2004). The Val^158^Met polymorphism has been associated with cognitive and emotional phenotypes (e.g. Egan et al., 2001; Mier et al., 2010), as well as dopamine-modulated psychiatric disorders (Egan et al., 2001; Pooley et al., 2007). These studies have generally demonstrated that the low activity Met^158^ allele of COMT, presumed to result in increased prefrontal dopamine levels, is associated with enhanced cognitive function (Barnett et al., 2007, though also see Barnett et al., 2008; Egan et al., 2001) and more efficient neural processing (Egan et al., 2001; Mier et al., 2010), compared with the Val^158^ allele. Studies in rodents have consistently supported these findings by demonstrating that lower COMT activity, mediated either genetically (Babovic et al., 2008; Papaleo et al., 2008) or pharmacologically with the centrally-acting COMT inhibitor, tolcapone (Lapish et al., 2009; Tunbridge et al., 2004), predicts superior performance on cognitive tasks, compared to wild-type or vehicle-treated animals which have high COMT activity.

Most existing studies ascribe the beneficial effects of low COMT activity on cognitive performance to effects in the prefrontal cortex (Egan et al., 2001; Tunbridge et al., 2004; Weinberger et al., 2001). However, COMT’s impact in other brain regions remains largely unexplored, and might also contribute to these associations. The hippocampus is a promising candidate region in this regard because of its involvement in learning and memory. The hypothesis that the hippocampus might, at least in part, be involved in mediating COMT’s links with cognitive function is attractive, since COMT is highly expressed in this region (Matsumoto et al., 2003) and the Val^158^Met polymorphism is associated with the activation of the hippocampus, and its functional connectivity with the prefrontal cortex, during human cognitive performance (Bertolino et al., 2006; Schott et al., 2006).

In order to investigate whether COMT might impact on hippocampal function, we examined the effects of the COMT inhibitor tolcapone on hippocampal neurochemistry and hippocampus-dependent cognitive performance. We focussed on the dorsal hippocampus, since it has a preferential role in spatial learning and memory, compared with the ventral hippocampus (Bannerman et al., 1999; Bannerman et al., 2004; Hock and Bunsey, 1998; Moser and Moser, 1998; Moser et al., 1995) . Tolcapone altered dorsal hippocampal dopamine neurochemistry (increasing dihydroxyphenylacetic acid (DOPAC) and reducing homovanillic acid (HVA)) and improved performance on the dorsal hippocampal-dependent delayed, rewarded alternation and spatial novelty preference tasks. Our data suggest that, along with its action in the prefrontal cortex, COMT’s function in the hippocampus might contribute to its association with cognition.

## Materials and methods

### Animals

Lister Hooded rats (Harlan-Olac, Bicester, UK), housed in groups of three under standard conditions (lights on 7.30–19.30, 21 ± 1°C temperature, 50% humidity, *ad libitum* water), were used. Male rats were used for all studies, and their weights were 217 ± 10 g (mean ± SD) for *ex vivo* neurochemistry, 233 ± 10 g for microdialysis and 263 ± 19 g for behavioural testing. Female rats (145 ± 8 g, age-matched to respective male group) were used only for *ex vivo* neurochemistry. Food was available *ad libitum*, apart from the rewarded alternation task, for which rats were maintained on a restricted feeding schedule at no less than 85% of their free-feeding body weight for 27–30 days prior to testing. All animal procedures were carried out in accordance with the Animals (Scientific Procedures) Act 1986 and associated Home Office guidelines.

### Drugs

Tolcapone (Roche Products Ltd, Welwyn, UK), a COMT inhibitor with no effect on other enzymes involved in synthesis or metabolism of amines (Zürcher et al., 1990), was suspended in 0.9% saline with a few drops of Tween-80 and administered intraperitoneally at a dose of 30 mg/kg. This dose significantly inhibits COMT activity (Acquas et al., 1992) and has been shown to increase prefrontal dopamine levels and improve prefrontal-dependent cognitive function (Tunbridge et al., 2004). Furthermore, we have shown that this dose robustly inhibits COMT activity throughout the rat brain (Laatikainen et al., 2009). For behavioural testing, tolcapone or vehicle was administered 1 hour prior to testing and the experimenter was blind to treatment.

### Determination of catecholamines and dopamine metabolites in dorsal hippocampal homogenates

Rats (*n*=5–6 per group) were sacrificed by decapitation and the dorsal half of the hippocampus was rapidly dissected and snap frozen 2 h after drug administration. The tissue was homogenised in 0.06 M perchloric acid (Sigma-Aldrich Company Ltd, Dorset, UK) and centrifuged at 15000 rpm for 10 min at 4^o^C. Samples were separated using a Microsorb C_18_ column (100 x 4.6 mm column; 3 μm C_18_ Microsorb particles; Varian Inc, Oxford, UK) immediately after preparation. Neurochemicals were eluted at a flow rate of 1 mL/min with a 0.12 M NaH_2_PO_4_.H_2_O buffer solution at pH 3.3 containing 16% (v/v) methanol, 3 mM 1-octanesulphonic acid (OSA) and 1.07 mM EDTA. Tissue levels of dopamine, noradrenaline, HVA and DOPAC levels were electrochemically detected (BAS LC-48 amperometric detector, BAS Instruments) using a glassy carbon electrode working at +0.7 V (vs. a Ag/AgCl reference electrode; BAS Instruments, Kenilworth, UK).

### Microdialysis

Intracerebral MAB 6.14.IC guide cannulas (Royem Scientific Ltd, Luton, UK) were stereotactically implanted above the dorsal hippocampus and were secured in position with Refobacin bone cement R (Biomet Europe, Germany) under isoflurane anaesthesia. After surgery, rats (*n*=5–6 per group) were administered buprenorphine (0.3mg/kg i.m.; Vetergesic; Alstoe Ltd, Sheriff Hutton, York, UK) and housed in pairs in recovery cages. Following a minimum 3-day recovery period, concentric MAB 6.14.3 microdialysis probes (3 mm PES dialysis membrane with 0.6 mm diameter; Royem Scientific Ltd, Luton, UK) were inserted into the dorsal hippocampus (AP -4.3, ML -2.5, DV -4 relative to bregma and dura surface (Paxinos and Watson, 1997)) under light isoflurane anaesthesia. The microdialysis probe was perfused at a rate of 2 μL/min with artificial cerebrospinal fluid (aCSF: 140 mM NaCl, 3 mM KCl, 1.2 mM Na_2_HPO_4_.2H_2_O, 0.27 mM NaH_2_PO_4_.1H_2_O, 1 mM MgCl_2_.6H_2_O, 2.4 mM CaCl_2_ and 7.2 mM D-glucose) by a CMA/100 perfusion pump (CMA/100, Carnegie Medicine, Stockholm, Sweden) via a liquid swivel system for freely moving animals (Instech Laboratories Inc, Plymouth, PA, USA). Perfusate samples were collected every 20 min and were separated using a Microsorb C_18_ column immediately after collection. Neurochemicals were eluted at a flow rate of 1 mL/min with a 0.12 M NaH_2_PO_4_.H_2_O buffer solution at pH 2.8–4.3 containing 16% (v/v) methanol, 0.5–4.9 mM 1-octanesulphonic acid (OSA), 1.07 or 100 mM EDTA and 2 mM NaCl. Extracellular levels of HVA and DOPAC were detected using an ANTEC Decade II amperometric detector (set at 35–45°C, Antec Leyden, Zoeterwoude, The Netherlands), which was equipped with an ISAAC flow cell (Antec Leyden, Zoeterwoude, The Netherlands) operated at +0.6 -0.65 mV. Once basal neurochemical levels had stabilised, tolcapone or vehicle was administered and dialysates were collected and analysed for an additional 2 h. Once the experiment was complete, the brain was removed and frozen, and probe placement was verified histologically.

### Delayed rewarded alternation

Delayed, rewarded alternation (McHugh et al., 2008; Rawlins and Olton, 1982) was assessed in a single cohort of experimentally naïve male rats (*n*=8 per group). The apparatus consisted of an open wooden T-maze, raised 43 cm off the floor (arms 61 x 10 x 2 cm) and painted a uniform grey colour. Each Goal arm contained a metal food well (2 cm in height and 2.5 cm in diameter), which could be baited with a food reward (45 mg Noyes sucrose pellets, Sandown Scientific, UK), and was located 5 cm from the distal end of the arm. A movable block of wood (natural wood colour, 20 x 10 x 10 cm) could be placed on the maze to deny access to one of the Goal arms.

Rats were habituated to the maze and the food reward. During rewarded alternation testing, each trial consisted of a sample run and a choice run. During the sample phase, rats were trained to run from the Start arm to the only accessible Goal arm, which was baited with one sucrose pellet. During training, the sample phase was immediately followed by the choice phase, during which rats were rewarded for choosing the novel Goal arm by the presence of two sucrose pellets in this arm. The previously visited Goal arm had no food available during the choice phase. After the criterion of 80% alternation was reached, a delay of either 30 s or 600 s was introduced between the sample phase and the choice phase. During delays, rats were placed in 30 x 12.5 x 15 cm wooden holding cages with a wire-mesh floor and a transparent Perspex lid. The order of delays and the location of the rewarded Goal arm were varied in a pseudorandom fashion between trials within each block of ten trials, with no more than two consecutive sample runs with the same configuration.

On each of the four testing days six animals were tested, of which two were drug-treated, two were vehicle-treated and two were non-injected controls. Each rat received 4 blocks of 10 trials on the testing day, and thus received 20 trials with a 30 s delay and 20 trials with a 600 s delay. The latency to eat on the sample run was also recorded.

### Spatial novelty preference

Spatial novelty preference (Sanderson et al., 2007) was assayed in 2 separate cohorts of experimentally-naïve male rats tested 1 month apart in separate testing rooms (final *n*=20–21 per group). Behavioural testing was performed in batches of six individually running rats, of which two were drug-treated, two were vehicle-treated and two were simply handled (non-injection controls). The apparatus used was a Y-maze constructed from transparent Perspex, which was mounted on a transparent Perspex board (47.5 x 52.5 cm). The walls of the maze were 11.5 cm high and 0.3 cm thick. Each arm was 20 cm long and 8 cm wide, and filled with a thin layer (<0.5 cm) of wood chips. Each trial consisted of 2 phases, a 5 min exploration phase and a 2 min test phase, separated by a 1 min delay. During the exploration phase rats could explore the Start arm and the Other arm. Access to the remaining arm of the Y-maze (the Novel arm) was blocked with a sheet of opaque Perspex. During the test phase rats could freely access all three arms of the Y-maze. Timing of each phase was started when the rat left the Start arm by placing all four paws outside the arm. The main outcome measure was the time spent in each arm; the number of entries into each arm was also recorded. Allocation of arms (Start, Other and Novel) to specific spatial locations and to the arms of the Y-maze was counterbalanced within each experimental group.

### Data analysis

Data were analysed using IBM SPSS Statistics v19. None of the data required transformation before analysis. Hippocampal homogenate data were analysed by two-way ANOVA, with LSD post-hoc tests, with drug treatment and sex as between-subjects factors. Microdialysis data were expressed as a percentage of baseline (calculated as the mean of the three samples prior to drug administration) and were analysed using repeated-measures ANOVA, with time as the repeat factor and drug treatment as a between-subjects factor. Greenhouse-Geisser corrections were applied where data failed Mauchly’s test of sphericity. For delayed rewarded alternation, the choice data from a given delay were combined into 1 block of 20 trials for each delay for each rat. They were then analysed by repeated-measures ANOVA, with delay (30 or 600 sec) as the within-subjects factor and treatment as a between-subjects factor. Novelty preference was quantified by calculating a discrimination ratio [Novel/(Novel+Other)] for the time spent in each arm during the test phase, and analysed using a one-way ANOVA with treatment as the between-subjects factor. Including rat cohort as a between-subjects factor had no statistically significant effect on novelty preference and hence was not included as part of the final analysis (its inclusion or exclusion did not change the overall findings; data not shown).

## Results

### Effect of tolcapone on tissue levels of dopamine, noradrenaline, DOPAC and HVA in the hippocampus

**Figure 1.**
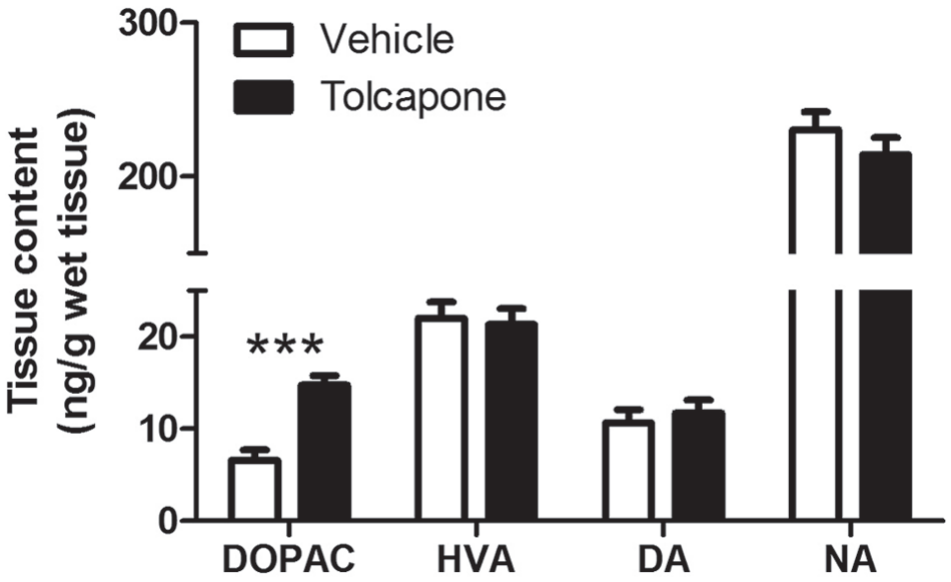
The effect of COMT inhibition on tissue neurochemical levels. Levels (ng/g wet tissue) of DOPAC, HVA, dopamine (DA) and noradrenaline (NA) in the dorsal hippocampus in vehicle- (open bars; pooled data from *n* = 6 males + 5 females) and tolcapone-treated (closed bars; pooled data from *n* = 6 males + 6 females) rats are shown. Bars indicate means ± SEMs. *** *p*<0.001.

Tolcapone-treated rats had higher tissue levels of DOPAC (F_1,19_=29.0; *p*<0.001), but not HVA, dopamine or noradrenaline (Fs<1.0, *p*s>0.1), compared with vehicle-treated rats (Figure 1). Drug treatment did not significantly alter the noradrenaline/dopamine ratio (F<1; *p*>0.1), which was 24.9 ± 3.0 in the vehicle group and 21.0 ± 2.9 in the tolcapone-treated animals (means ± SEM). There were no main effects of sex, nor sex*treatment interactions (Fs<1.5, *p*s>0.5) on any of the measures, except that males rats had significantly higher noradrenaline levels in the dorsal hippocampus, compared with females (F_1,19_=5.6, *p*=0.028).

### Effect of tolcapone on extracellular levels of dopamine metabolites in the dorsal hippocampus

**Figure 2.**
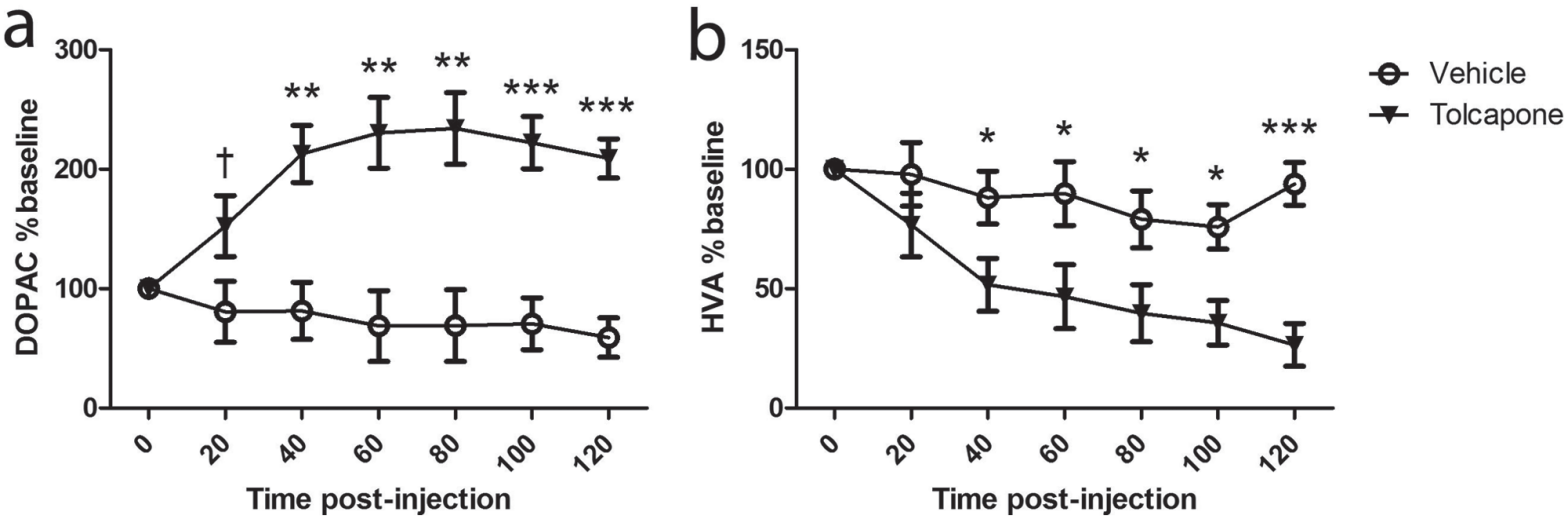
The effect of COMT inhibition on extracellular (a) DOPAC and (b) HVA. Tolcapone treatment (closed triangles) significantly increased (a) DOPAC (*n*=5 per group) and decreased (b) HVA (*n*=6 per group), compared with vehicle-treated animals (open circles). Data shown are expressed as a percentage of baseline, and given as means ± SEMs. ^†^*p*<0.1; *p<0.05; **p<0.01; *** *p*<0.001.

Extracellular DOPAC and HVA were altered by COMT inhibition. For both DOPAC and HVA, there were significant main effects of time (HVA: F_6,60_=8.0; *p*<0.001; DOPAC: F_2.4,18.9_=2.7; *p*<0.1 [significant at trend level only]) and treatment (HVA: F_6,60_=3.2; *p*<0.01; DOPAC: F_2.4,18.9_=6.6; *p*=0.005), and significant time*treatment interactions (HVA: F_1,10_=11.2; *p*<0.01; DOPAC: F_1,10_=24.2; *p*=0.001). These effects were due to an increase in DOPAC and decrease in HVA from baseline in the tolcapone-treated animals, which in both cases was significant from 40 minutes post-injection to the end of the experiment (*p*s<0.01; Figure 2). Dopamine and noradrenaline could not be reliably detected in hippocampal dialysates.

### Effect of tolcapone on dorsal hippocampus-dependent cognitive task performance

Tolcapone-treated male rats performed significantly better than vehicle-treated rats on the delayed, rewarded alternation and spatial novelty preference tasks, with non-injected rats showing intermediate levels of performance.

**Figure 3.**
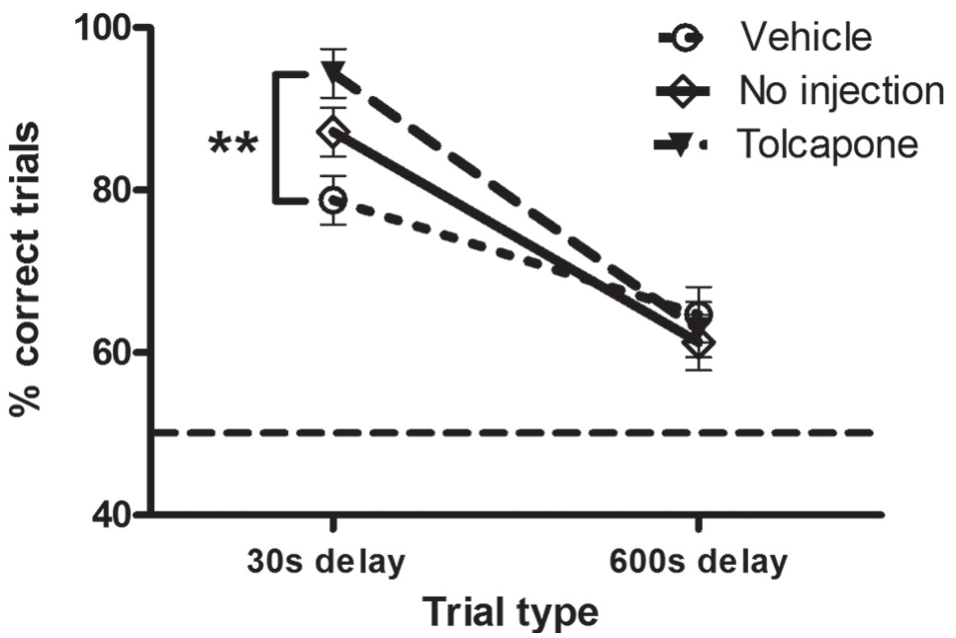
The effect of COMT inhibition on delayed, rewarded alternation. Performance (percentage of correct trials; means ± SEMs of 20 trials) at 30 and 600 second delays in vehicle- (open circles, dotted line; *n* = 8) and tolcapone-treated (closed triangles, dashed line; *n* = 8) rats, and non-injected controls (open diamonds, solid line; *n* = 8). The dashed line indicates chance performance (50% correct). **p<0.01.

A repeated-measures ANOVA investigating performance on the rewarded alternation task (with a between-subjects factor of treatment and a within-subjects factor of delay) showed a significant treatment*delay interaction (F_2,21_=4.8, *p*=0.020) and a main effect of delay (F_1,21_=102.1, *p*<0.001), but no main effect of treatment (F_2,21_=2.0, *p*=0.163). Post-hoc tests revealed that tolcapone-treated rats made more correct choices compared with vehicle-treated rats during the 30 s delay condition (*p*=0.001; Figure 3). In contrast, task performance after a 600 s delay was similar across the three treatment groups. Not surprisingly, performance levels were much lower on trials with a 600 s delay but were still significantly above chance (50 %; t(23)=6.8, *p*<0.001). Interestingly, non-injected rats tended to perform better than vehicle-treated rats on the 30s delay condition (*p*=0.060). Analysis of the latencies to eat on the sample phase across different trial delays during testing did not show any effect of treatment or delay*treatment interaction (Fs<0.7, *p*s>0.05 during training and testing).

It is important to note that the three groups of rats did not differ in their performance levels during the pre-training stage, prior to any drug administration. There was no overall effect of ‘future’ treatment (F_2,21_=0.5, *p*=0.635) or delay*treatment interaction (F_2,21_=0.9, *p*=0.426) on baseline performance during pre-training (i.e. prior to injection; data not shown).

**Figure 4.**
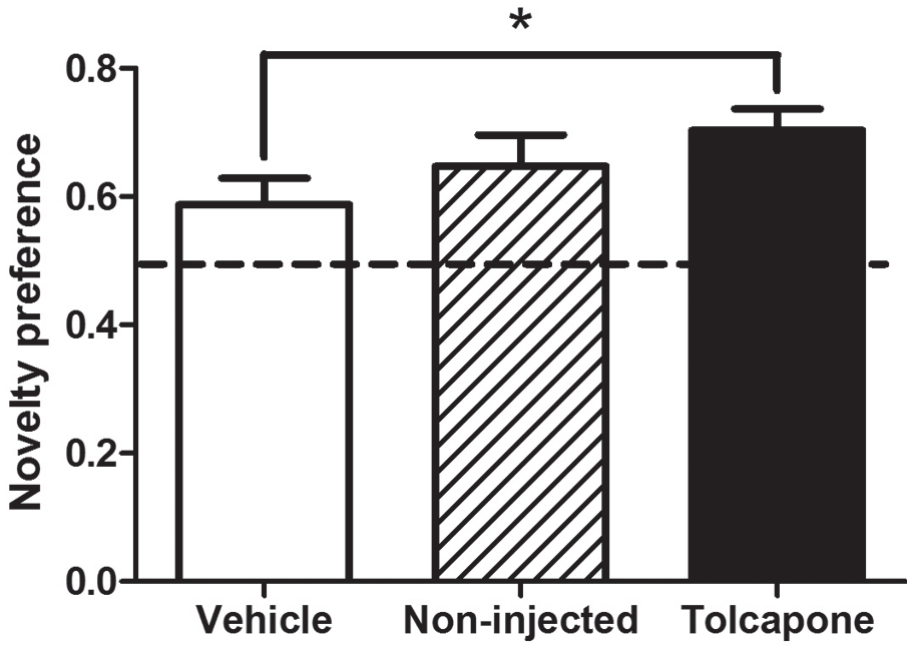
The effect of COMT inhibition on spatial novelty preference. Novelty preference was significantly increased in tolcapone-treated rats (closed bar; *n*=20), compared with those vehicle-treated (open bars; *n*=20). Non-injected controls (cross-hatched bars; *n*=21) showed intermediate levels of performance. Bars indicate means ± SEMs; chance performance (50%) is indicated by the dashed line. *p<0.05.

During the spatial novelty preference task, similar to the rewarded alternation task, tolcapone-treated animals showed significantly greater spatial short-term memory (in terms of spatial novelty preference), relative to vehicle-injected controls, with non-injected animals again demonstrating intermediate levels of novelty preference. A one-way ANOVA with treatment as a between-subjects factor did not yield an overall main effect of treatment (F_2,58_=1.9, *p*=0.166). However, based on the results of the rewarded alternation experiment, in which there was a significant difference between tolcapone- and vehicle-treated rats, we performed an *a priori* planned comparison (Howell, 1997). This analysis showed that tolcapone-treated rats show greater novelty preference than vehicle-treated rats (Figure 4; F_1,38_=4.7, *p*=0.037).

Given that Maj et al. (1990) found that 30 mg/kg tolcapone increased exploratory activity in rats, it is possible that the tolcapone-induced enhancement of novelty preference might reflect increased exposure to the open arm (destined to be the Other arm) during the exploration phase of the task, thereby making this arm more familiar to the animal. However, this did not appear to be the case: treatment had no effect on the time spent investigating the open (Other) arm during the 5 min exploration period (F_2,58_=0.04, *p*=0.957), arguing against an effect of COMT inhibition on exploration *per se*. Also, the number of entries into each arm during the exploration phase did not differ between treatment groups (Fs<1)(data not shown).

## Discussion

We have shown that COMT inhibition alters dorsal hippocampal catecholamine metabolism, both *ex vivo* and *in vivo*, and enhances performance on dorsal hippocampus-dependent cognitive tasks. Thus, our data suggest direct effects of COMT on catecholamine function in the dorsal hippocampus that might contribute to its modulation of cognitive function.

### Tolcapone affects catecholamine metabolism in the dorsal hippocampus

Acute COMT inhibition with tolcapone increased DOPAC in dorsal hippocampal tissue extracts and dialysate samples. Furthermore, tolcapone also reduced HVA levels in dorsal hippocampal dialysates (which reflect the extracellular pool of HVA) but not in tissue extracts (which include all pools of HVA). We consider that this apparently differential effect of tolcapone on HVA measured using microdialysis vs. *ex vivo* is most likely the result of the relatively greater power of the microdialysis study to detect a difference (since baseline variations in HVA levels could be corrected for), compared to the tissue study (in which baseline correction was not possible). However, it is also possible that COMT activity is particularly important for regulating the extracellular pool of HVA in the hippocampus.

Even though central dopamine metabolism has been extensively studied both *ex vivo* and *in vivo*, the present study is the first investigation of the neurochemical effects of COMT inhibition on dopamine levels in the dorsal hippocampus (although Li et al. (1998) examined its effects on noradrenaline and its metabolites). The findings of this paper mirror previous neurochemical findings using tolcapone in the frontal cortex and striatum (e.g. Maj et al., 1990; Tunbridge et al., 2004), in that tolcapone administration increased DOPAC levels and decreased HVA levels. Furthermore, our finding that COMT modulates hippocampal dopamine metabolism is consistent with its high expression in this region (Matsumoto et al., 2003).

Although we have found that COMT inhibition modulates levels of the dopamine metabolites DOPAC and HVA, caution should be exercised when interpreting our findings as reflecting an effect of COMT on hippocampal dopaminergic transmission, since DOPAC originates in part from noradrenergic neurons in this region (Vahabzadeh and Fillenz, 1992). Noradrenaline is substantially more abundant than dopamine in the hippocampus (we found a noradrenaline/dopamine ratio of ~23, as detailed above), although it is now clear that the hippocampus does receive a specific, albeit relatively sparse, dopaminergic innervation (Gasbarri et al., 1997). Thus, although in the frontal cortex COMT impacts specifically on dopamine, and not noradrenaline (Tunbridge et al., 2004), further studies are required to investigate whether this selective effect also pertains in the hippocampus.

### Tolcapone improves dorsal hippocampus-dependent memory

We found that rats given tolcapone showed better spatial short-term memory performance, compared with vehicle-treated rats, with non-injected rats performing at intermediate levels. This apparently cognitive enhancing effect of tolcapone was seen both in the delayed alternation and spatial novelty preference tasks. Our results are consistent with previous preclinical studies using tolcapone, which have shown that lowering COMT activity has beneficial effects on certain aspects of cognitive function, including medial prefrontal cortex-dependent extradimensional set shifting (Tunbridge et al., 2004) and delayed spatial win-shift performance (Lapish et al., 2009), and (albeit Val^158^Met genotype-dependent) improvements in cognitive function seen after tolcapone administration in humans (Apud et al., 2007; Farrell et al., 2012; Gasparini et al., 1997; Giakoumaki et al., 2008; Roussos et al., 2009).

Our spatial novelty preference test findings cannot be explained by effects of tolcapone on the extent to which rats explored the Other arm during the sample phase. Neither can our results be due to COMT inhibition impacting on proactive interference, since tolcapone improved performance on the spatial novelty preference task in which animals complete only a single trial. It is also worth pointing out that tolcapone was effective in enhancing cognitive performance on the spatial novelty preference task in which there is no overt reward or reinforcement (of relevance given effects of COMT on certain reward-related phenotypes (Tunbridge et al., 2012)). However, one explanation for the tolcapone-induced cognitive enhancement may involve an increase in the extent of short-term habituation to recently experienced, familiar environments (Sanderson and Bannerman, 2010; Sanderson et al., 2007). Indeed, we have argued previously that the rewarded alternation (win-shift) maze task and the spatial novelty preference task may be similar in that they both provide a measure of how well rodents detect and perceive relative novelty/familiarity. Our data are consistent with this hypothesis, in that the tolcapone-induced cognitive enhancement seen in this study could be due to an increase in short-term habituation to familiar environments.

One puzzling aspect of our results is the observed treatment*delay interaction on the delayed rewarded alternation task, i.e. that tolcapone enhanced performance after a 30 s but not a 600 s delay. Our finding that tolcapone also improved performance on the spatial novelty preference task makes it unlikely that the improvement seen after the 30 s delay is a false positive. Although rats performed considerably more poorly on the 600 s than the 30 s delay condition, their performance was still above chance levels. Nevertheless, the scope for observing an effect of COMT inhibition may be dependent on the animals’ baseline performance levels, which in turn could be influenced by the temporal demands of the task. However, given that Lapish et al. (2009) also found that tolcapone generally improved performance on a hippocampus-dependent task under conditions of extended delay (3 hours), it appears that the temporal dynamics of the cognitive enhancing effects of COMT might be very subtle and task parameter-dependent (Lisman et al., 2011).

A second surprising aspect of our results is that vehicle injected animals performed more poorly, compared to the non-injected controls on both the rewarded alternation and spatial novelty preference tasks. This finding stands in contrast to the relatively better performance (albeit non-significantly so) seen in the tolcapone-treated animals, when compared to the non-injected controls. This relative impairment in the vehicle-treated animals is perhaps surprising, as it might be anticipated that the elevation in dopamine transmission expected to result from injection stress (Yamato et al., 2002) would translate into an improvement in behavioural performance in vehicle-injected animals, compared to the non-injected controls. However, as well as increasing dopamine, stress modulates the function of multiple neurotransmitter systems and signalling cascades in the hippocampus (Joca et al., 2007; Yamato et al., 2002). Therefore, we believe the most parsimonious explanation for our results is that vehicle administration impairs behavioural performance, compared to non-injected controls, by non-catecholaminergic mechanisms and that the cognitive enhancing effects of tolcapone (presumed to be mediated via an increase in catecholaminergic transmission) are superimposed on top of this vehicle administration-induced impairment. The trend-level significant results in the non-injected group will need to be validated and further studied in the future; nevertheless, our findings emphasise the need to include a non-injected control group in studies of this nature.

### The role of the hippocampus in mediating the effects of tolcapone on cognition

Our results do not speak directly to the functional consequences of the neurochemical changes induced by COMT inhibition in the dorsal hippocampus, since tolcapone was administered systemically, and therefore presumably inhibited COMT throughout the brain. However, our findings of an effect of COMT inhibition on dorsal hippocampal dopamine metabolism raise the possibility that COMT acting directly in this region could mediate tolcapone’s effects on dorsal hippocampus-dependent cognitive function. This hypothesis is attractive on theoretical grounds given that hippocampal dopamine function modulates memory performance in humans (e.g. Schott et al., 2006; Wittmann et al., 2005) and rodents (Goto and Grace, 2008; Packard and White, 1989), although it is also possible that the behavioural effects of tolcapone relate to effects on noradrenaline, or via other mechanisms entirely (Tunbridge et al., 2008).

Both delayed rewarded alternation and spatial novelty preference are hippocampus-dependent tasks (Deacon et al., 2002; Howland et al., 2008; Mumby et al., 2002; Sanderson et al., 2007). Of course we cannot rule out a contribution to these tolcapone effects from extra-hippocampal brain regions, including the prefrontal cortex, which is critical for multiple aspects of cognitive function and in which COMT has been shown to regulate dopamine function (Lapish et al., 2009; Tunbridge et al., 2004). However, medial prefrontal cortex (mPFC) lesions have been reported to have either no effect on alternation behaviour (Aggleton et al., 1995; Deacon et al., 2003), or to result in only mild and transient impairments ((Delatour and Gisquet-Verrier, 2000, Dias and Aggleton, 2000; Shaw and Aggleton, 1993) see Sanderson and Bannerman (2010) and Sanderson et al. (2010) for a full discussion of the relative importance of the hippocampus and prefrontal cortex for spatial alternation). Furthermore, previous studies suggest that the mPFC is not involved in spatial novelty preference *per se*, since its inactivation with lidocaine does not affect spatial recognition memory when assessed using this simple version of the spatial novelty preference task (Hannesson et al., 2004). Accordingly, it seems unlikely that the behavioural effects of tolcapone seen in this study (and in particular those observed on the spatial novelty preference task) can be entirely attributed to COMT’s impact on medial prefrontal dopamine. Furthermore, our data raise the possibility that some of the effects of tolcapone previously ascribed to its effects on prefrontal dopamine (Lapish et al., 2009) might be, at least in part, mediated by its impact on hippocampal catecholamine function.

In conclusion, we have demonstrated that COMT inhibition improves the performance of hippocampal-dependent memory tasks in rats. Furthermore, we have also shown that COMT inhibition impacts on dopamine metabolism in the dorsal hippocampus, suggesting that direct effects of COMT in the dorsal hippocampus might underlie our observed behavioural effects. These findings provide additional support for the hypothesis that pharmacologically reducing COMT activity might be a useful therapeutic approach for treating cognitive dysfunction in disorders such as schizophrenia, albeit potentially dependent on COMT genotype (Chen et al., 2011; Farrell et al., 2012). Moreover, our results suggest that, in addition to the prefrontal cortex, the dorsal hippocampus could be a key brain region for mediating COMT’s impact on cognitive function. This proposal is consistent with reported associations between COMT and episodic memory performance in humans (Bertolino et al., 2006; de Frias et al., 2004).
